# Functional evidence that Activin/Nodal signaling is required for establishing the dorsal-ventral axis in the annelid *Capitella teleta*

**DOI:** 10.1242/dev.189373

**Published:** 2020-09-23

**Authors:** Alexis R. Lanza, Elaine C. Seaver

**Affiliations:** Whitney Laboratory for Marine Bioscience, University of Florida, 9505 Ocean Shore Boulevard, St Augustine, FL 32080-8610, USA

**Keywords:** TGF-β, BMP, Morpholino, Annelid, Axes formation, Spiralian

## Abstract

The TGF-β superfamily comprises two distinct branches: the Activin/Nodal and BMP pathways. During development, signaling by this superfamily regulates a variety of embryological processes, and it has a conserved role in patterning the dorsal-ventral body axis. Recent studies show that BMP signaling establishes the dorsal-ventral axis in some mollusks. However, previous pharmacological inhibition studies in the annelid *Capitella teleta*, a sister clade to the mollusks, suggests that the dorsal-ventral axis is patterned via Activin/Nodal signaling. Here, we determine the role of both the Activin/Nodal and BMP pathways as they function in *Capitella* axis patterning. Antisense morpholino oligonucleotides were targeted to *Ct-Smad2/3* and *Ct-Smad1/5/8*, transcription factors specific to the Activin/Nodal and BMP pathways, respectively. Following microinjection of zygotes, resulting morphant larvae were scored for axial anomalies. We demonstrate that the Activin/Nodal pathway of the TGF-β superfamily, but not the BMP pathway, is the primary dorsal-ventral patterning signal in *Capitella*. These results demonstrate variation in the molecular control of axis patterning across spiralians, despite sharing a conserved cleavage program. We suggest that these findings represent an example of developmental system drift.

## Abstract (precis)

**Summary:** Morpholino knockdown experiments in the annelid *Capitella teleta* demonstrate that the dorsal-ventral axis is primarily patterned by the Activin/Nodal pathway of the TGF-β superfamily, rather than by the BMP pathway.

## INTRODUCTION

**Fig. 1.**
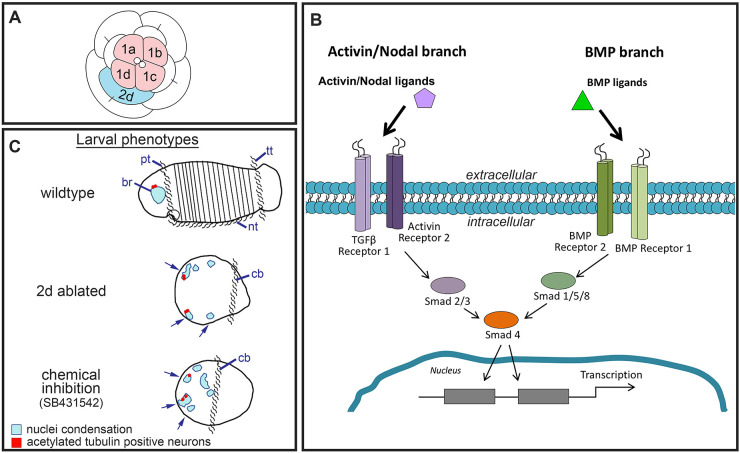
**Schematics of spiralian embryo, TGF-β superfamily cassette and larval *Capitella* phenotypes.** (A) *Capitella* organizer cell 2d is shown in blue, first quartet cells are in pink. (B) General schematic depicting both branches of the TGF-β superfamily pathway and the key signaling components. Arrows indicate condensation of nuclei. (C) Phenotypes seen in stage 6 *Capitella* larvae following perturbations. br, brain; cb, ciliary band; nt, neurotroch; pt, prototroch; tt, telotroch.

The body axes of many animals are patterned during embryonic development via a cell signaling center known as an organizer (Amiel et al., 2013; Clement, 1962, 1976; Gerhart et al., 1991; Goldstein and Freeman, 1997; Kimmel et al., 1990; Kraus et al., 2016; Martindale, 1986; Onai et al., 2010; Shih and Fraser, 1996). In spiralians, a large bilaterian clade that exhibits enormous body plan diversity, embryos share a highly stereotypic early development program called spiral cleavage, and signals emanating from single cells during early cleavages are crucial for patterning the dorsal-ventral axis (Amiel et al., 2013; Clement, 1962; Damen and Dictus, 1996a; Henry and Perry, 2008; Henry et al., 2006; Lambert and Nagy, 2003). In the spiralian annelid *Capitella teleta*, the first two cleavage divisions in the embryo are unequal and produce four blastomeres named A, B, C and D. Their descendants subsequently define the four quadrants of the embryo (Conklin, 1897). The A, B, C and D blastomeres, also known as macromeres, divide to produce multiple sets of quartets of smaller daughter cells, called micromeres. Lineage tracing and single-cell ablation studies have demonstrated that micromere 2d (Fig. 1A, blue cell) generates ectoderm of the larval trunk and pygidium in *Capitella* and that the presence of 2d is necessary for establishing bilateral symmetry and dorsal-ventral organization of the larval head (Amiel et al., 2013; Carrillo-Baltodano and Meyer, 2017). Head structures are derived from descendants of the first quartet micromeres 1a, 1b, 1c and 1d (Fig. 1A, pink cells), not cell 2d (Carrillo-Baltodano and Meyer, 2017; Meyer and Seaver, 2010; Meyer et al., 2010). Cell 2d functions as an organizer and induces the head precursor cells via an inductive signal that is required through the 16-cell stage (Amiel et al., 2013). Chemical inhibition investigations into the identity of the molecular signal utilized by cell 2d to orchestrate axis specification in *Capitella* suggest that signaling occurs via the Activin/Nodal pathway (Lanza and Seaver, 2018). Interestingly, in a few other spiralians, dorsal-ventral patterning occurs via the BMP signaling pathway (Kuo and Weisblat, 2011; Lambert et al., 2016; Tan et al., 2017, 2018).

The TGF-β superfamily signaling pathway regulates a myriad of developmental processes, including patterning of the dorsal-ventral axis (Wu and Hill, 2009). This superfamily comprises two distinct branches: the Activin/Nodal branch, activated by secreted ligands such as TGF-β, Nodal and Activin; and the BMP branch, activated by secreted ligands such as BMP5-8, BMP2/4 and ADMP (Fig. 1B). Previous analyses have identified all the components necessary for signaling via both branches in the *Capitella* genome (Kenny et al., 2014).

Using functional methods established for *Capitella* by Klann and Seaver (2019), this study uses antisense morpholino oligonucleotides (MOs) to demonstrate a role for Activin/Nodal signaling as it functions in *C. teleta* axes patterning, and demonstrates that BMP signaling does not play a primary role in patterning the dorsal-ventral axis. Morpholinos were targeted to *Ct-Smad2/3* and *Ct-Smad1/5/8*, receptor signal transducers specific to the Activin/Nodal and BMP pathways, respectively. We expected that morphants would phenotypically resemble larvae resulting from 2d deletion and chemical inhibition studies (Fig. 1C) (Amiel et al., 2013; Lanza and Seaver, 2018). Morphants were rescued by microinjection of *Ct-Smad2/3* mRNA. These results provide the first example of mRNA rescue in *C. teleta*.

## RESULTS

### Temporal changes and spatial expression of TGF-β superfamily transcripts

**Fig. 2.**
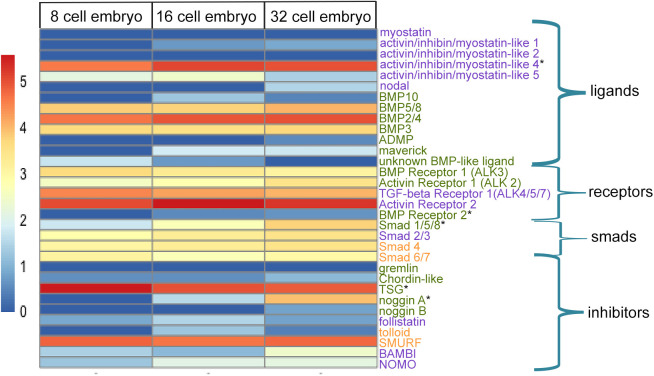
**Transcript counts of TGF-β superfamily signaling components in early cleavage stage embryos.** Heatmap shows the log10 values of relative transcript levels. Blue and red indicate low and high levels of transcript abundance, respectively. Each row represents the expression of a single gene. Each column represents an embryonic stage. Components listed in purple are associated with Activin/Nodal signaling, green with BMP signaling and orange with both branches. Components are grouped into functional categories: ligands, receptors, Smads and inhibitors. Asterisks indicate genes with differential expression.

**Fig. 3.**
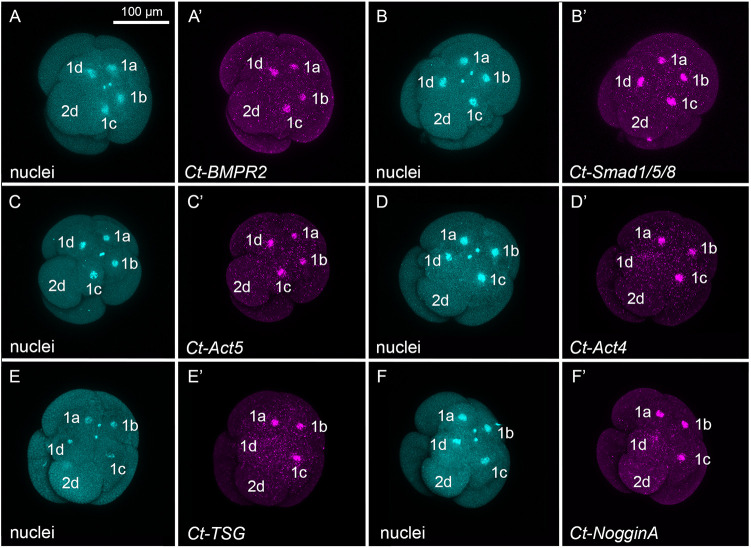
**Spatial localization of TGF-β pathway components.** (A-F′) Merged confocal stack images of embryos with organizer cell 2d present. Images with the same lettering correspond to a single embryo. Nuclei are labeled via anti-histone antibody (blue, A-F). Spatial localization of *Ct-BMP Receptor 2* (*BMPR2*), *Ct-Smad1/5/8*, *Ct-activin/inhibin/myostatin-like5 (Act5*), *Ct- activin/inhibin/myostatin-like4* (*Act4*)*, Ct-TSG* and *Ct-NogginA* are visualized by FISH (magenta, A′-F′). Expression is localized in and around the nucleus, as is occasionally seen in *C. teleta* early stage embryos (Boyle and Seaver, 2008; Boyle et al., 2014; Lanza and Seaver, 2018). 2d and first quartet cells are labeled. All genes are differentially expressed, except *Act5*.

Gene expression levels for TGF-β superfamily signaling components present in the *Capitella* genome were examined in single embryos at three time points: 8-cell, 16-cell (during organizer activity) and 32-cell (post-organizer activity) (Fig. 2). Transcript levels of each component in the Activin/Nodal and BMP pathways were examined to gain insight into the molecular identity of the organizing signal and identify pathway-specific candidates for MO knockdown. The transcripts detected confirm that the necessary components for both pathways are present and expressed during organizing activity (Fig. 2). In addition, fluorescent *in situ* hybridization (FISH) was conducted on embryos with organizer cell 2d present in order to characterize spatial patterns of differentially expressed genes and one additional Activin/Nodal ligand (Fig. 3; Table S1).

The *Capitella* genome possesses five TGF-β superfamily receptors (Fig. 2, receptors) – two associated with the Activin/Nodal family and three in the BMP family (Kenny et al., 2014). Across the three cleavage stages examined, the Activin/Nodal receptors *TGF-β Receptor 1* and *Activin Receptor 2* are expressed at higher levels relative to the three BMP family receptors. This suggests higher activity of the Activin/Nodal pathway than the BMP pathway during organizer activity. A previous investigation demonstrated that both Activin/Nodal receptors are expressed in first and second quartet micromeres at the 16-cell stage (Lanza and Seaver, 2018). Although formation of a heterodimer between type I and type II receptors is typically necessary for signaling, *BMP Receptor 2*, the only type II receptor in the *Capitella* genome associated with BMP signaling, is expressed at much lower levels than the *Capitella* Activin/Nodal type II receptor, *Activin Receptor 2*. *BMP Receptor 2* was detectable by FISH and was localized to first quartet micromeres in all four embryonic quadrants (Fig. 3A,A′). In other animals, ligand-receptor promiscuity occasionally occurs, whereby ligands bind with receptors of both the Activin/Nodal and BMP subfamilies and activate the respective ligand-family downstream Smads (Massagué, 2008; Mueller and Nickel, 2012). Due to the possibility that *Capitella* TGF-β receptors can bind ligands from both branches during early cleavages, we chose not to target receptors using MO knockdown strategies.

The *Capitella* genome contains four Smad genes: *Smad2/3* and *Smad1/5/8,* receptor-regulators of the Activin/Nodal and BMP branches, respectively; *Smad4*, a signal transduction mediator; and *Smad6/7*, an inhibitory receptor Smad (Kenny et al., 2014). Smad genes were moderately expressed during the cell stages examined with the exception of *Smad1/5/8*, whose relative expression was lower at the 8-cell stage and increased to moderate levels by the 16- and 32-cell stage (Fig. 2, smads). *Smad1/5/8* was typically expressed in all four first quartet micromeres in embryos with 2d present (Fig. 3B,B′). *Smad2/*3 was previously shown to be broadly expressed in first and second quartet micromeres (Lanza and Seaver, 2018). Although TGF-β superfamily receptors occasionally function via non-canonical (Smad-independent) pathways such as the MAPK pathway (Broege et al., 2013), previous chemical inhibition experiments demonstrated that MAPK is not involved in *Capitella* organizing activity (Amiel et al., 2013), demonstrating that signaling probably occurs via Smad-mediated signal transduction. Because *Smad2/3* and *Smad1/5/8* are specific to the Activin/Nodal and BMP branches, respectively, and both are expressed during organizer activity at the 16-cell stage, these genes were selected for MO knockdown.

The *Capitella* genome contains seven ligands that are transduced via the Activin/Nodal branch. Transcript levels of these Activin/Nodal ligands in the Activin/Nodal pathway were examined in an attempt to identify the ligand that mediates organizer activity (Fig. 2, ligands). *Myostatin* and *activin/inhibin/myostatin-like 2* were not transcribed during the time period examined (Fig. 2), and *activin/inhibin/myostatin-like 3* was not represented in the RNA-seq dataset. Similarly, *nodal* was not transcribed at the 8- or 16-cell stages, times associated with organizing activity. However, *nodal* was transcribed at low levels at the 32-cell stage, when the organizing activity signal is no longer required. *Activin/inhibin/myostatin-like 1*, *activin/inhibin/myostatin-like 4* and *activin/inhibin/myostatin-like 5* increased in relative transcript levels between the 8- and 16-cell stages (Fig. 2, ligands). Levels of *activin/inhibin/myostatin-like 5* decreased at the 32-cell stage. At the 16-cell stage, *activin/inhibin/myostatin-like 5* was expressed in all first quartet micromeres, whereas *activin/inhibin/myostatin-like 4* was expressed in a subset of first quartet micromeres, 1a, 1b and 1c (Fig. 3C-D′). These expression patterns do not resolve which, if any, of the Activin/Nodal ligands mediate organizing activity as it was expected that ligands functioning in organizing activity would be detected in micromere 2d.

Our expression data further indicate that several BMP ligands are transcribed during the time period examined. In particular, *BMP2/4, BMP5/8* and *BMP3* showed uniformly high transcript levels at all three time points. In addition, several inhibitors were transcribed (Fig. 2, inhibitors). Of these, *SMURF* , an intracellular inhibitor of receptor Smad transduction (Shi, 2001; Shi and Massagué, 2003), and *Twisted gastrulation* (*TSG*), an extracellular modulator of BMP signaling (Oelgeschläger et al., 2000; Ross et al., 2001), were transcribed at very high levels. Furthermore, transcript levels of *Noggin A*, an extracellular inhibitor of BMP ligands, significantly increased from the 8- to 16- and 16- to 32-cell stages. *TSG* and *Noggin A* were both expressed in the same first quartet micromeres, 1a, 1b and 1c, and were notably absent from D quadrant cells (Fig. 3E-F′).

### Morpholino knockdown of *Smad2/3* and *Smad1/5/8*

**Fig. 4.**
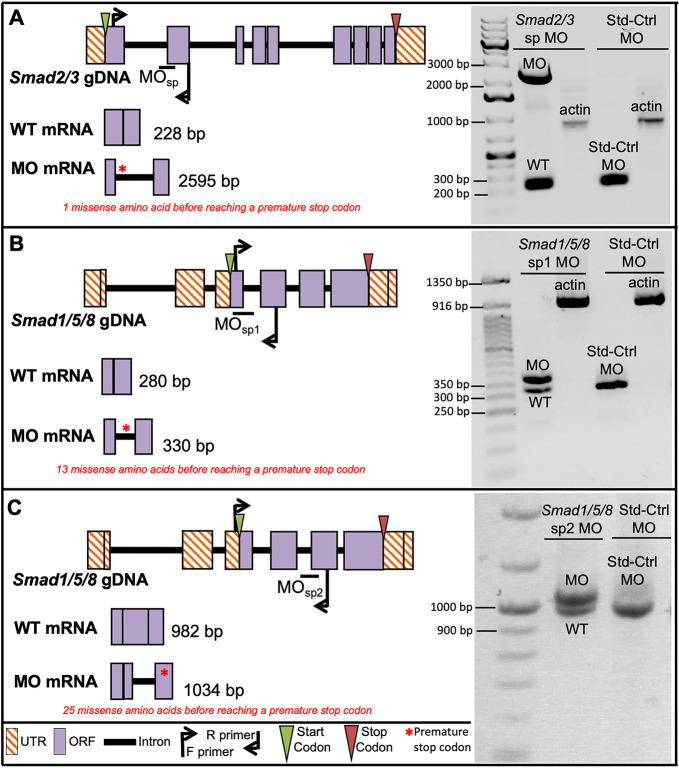
**Knockdown of *Smad2/*3 and *Smad1/5/8* using splice-blocking morpholinos.** (A) *Smad2/3* splice-blocking (sp) MO blocks splicing between intron 1 and exon 2 (top schematic). Middle schematic depicts expected size of wild-type (WT) transcripts. Bottom schematic depicts expected size of morphant transcripts. (B) *Smad1/5/8* sp1 MO blocks splicing between exon 3 and intron 3 (top schematic). Middle schematic depicts expected size of WT transcripts. Bottom schematic depicts expected size of morphant transcripts. (C) *Smad1/5/8* sp2 MO blocks splicing between intron 4 and exon 5 (top schematic). Middle schematic depicts expected size of WT transcripts. Bottom schematic depicts expected size of morphant transcripts. Red asterisks indicate position of premature stop codons resulting from MO splicing activity. Both WT and morphant transcripts were amplified from larvae injected as zygotes with *Smad2/3* sp MO (A), *Smad1/5/8* sp1 MO (B) and *Smad1/5/8* sp2 (C). Standard-control (Std-Ctrl) morphants yielded only WT-sized bands (A-C). Amplification of a 1000 bp actin fragment served as a cDNA quality control (A,B). Ladder band sizes are specified per gel image. ORF, open reading frame; UTR, untranslated region.

To assess the function of the Activin/Nodal and BMP signaling pathways during *Capitella* development, the pathway-specific transduction factors *Ct-Smad2/3* and *Ct-Smad1/5/8* were targeted using morpholino antisense oligonucleotides. A translation-blocking (tr) and a splice-blocking (sp) morpholino were designed to target *Smad2/3*, the *Activin/Nodal* receptor Smad (Fig. 4A). Splice blocking by *Smad2/3* sp MO results in the inclusion of intron 1 (Fig. 4A, bottom schematic) and introduces one missense amino acid before reaching a premature stop codon in the modified mRNA (Fig. 4A, red asterisk). Two non-overlapping sp morpholinos targeted *Smad1/5/8*, a BMP receptor Smad (Fig. 4B,C). The typical protein structure of receptor Smads consists of an N-terminal MH1 domain and a C-terminal MH2 domain. However, genomic (Simakov et al., 2012) and transcriptomic data indicate that *Ct-Smad1/5/*8 lacks an MH1 domain. Sufficient splice blocking of *Smad1/5/8* sp1 MO results in the inclusion of intron 3 (Fig. 4B, bottom schematic) and introduces 13 missense amino acids before reaching a premature stop in the modified post-spliced mRNA (Fig. 4B, red asterisk). Splice blocking of *Smad1/5/8* sp2 MO results in inclusion of intron 4 (Fig. 4C, bottom schematic), with a premature stop codon following 25 missense amino acids in the improperly spliced mRNA (Fig. 4C, red asterisk).

Microinjection of each of the three sp MOs resulted in the appearance of improperly spliced mRNAs (Fig. 4). PCR amplification of the *Smad2/3* sp targeted region confirmed the presence of a wild-type-sized band (228 bp) and a band of 2595 bp, which is the expected size if the first intron is retained following microinjection of zygotes with *Smad2/3* sp MO (Fig. 4A). Similarly, PCR amplification of *Smad1/5/8* sp1 and *Smad1/5/8* sp2 target regions resulted in both wild-type-sized bands (WT1, 280 bp; WT2, 982 bp) as well as bands that matched the size expected if the targeted introns are retained (sp1, 330 bp; sp2, 1034 bp) (Fig. 4B,C). In contrast, only wild-type-sized bands were observed following PCR of cDNA generated from larvae that had been injected with the standard control (Std-Ctrl) MO for all three primer sets (Fig. 4A-C). Together, these data confirm that all three sp MOs interfere with the proper splicing of their targeted pre-mRNAs and that the Std-Ctrl MO does not affect splicing of either *Smad2/3* or *Smad1/5/8*.

### Control morphant larvae phenotypically resemble wild type larvae

**Fig. 5.**
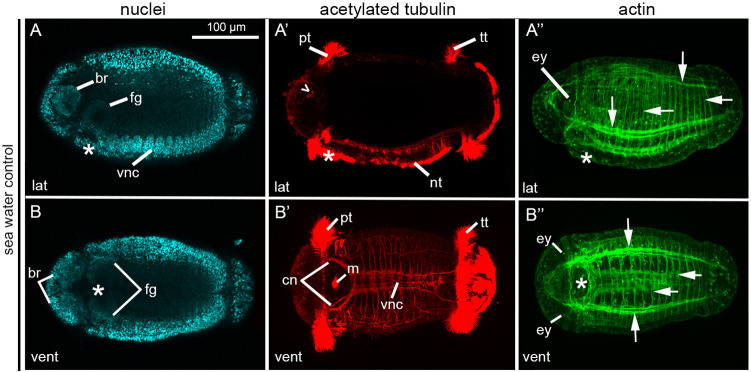
**Axial properties of wild-type *C. teleta* larvae.** (A-B″) Confocal projections of stage 6 larvae, oriented with anterior to the left. Columns show labeling of nuclei with Hoechst (A,B), cilia and neurons with anti-acetylated tubulin (A′,B′) and actin filaments with phalloidin (A″,B″). Larvae in A-A″ are in lateral view, B-B″ are in ventral view. Open arrowhead, sc^ac+^; asterisks, position of the mouth; white arrows, circumferential and longitudinal muscle fibers. br, brain; cn, circumoral nerve; ey, eye; fg, foregut; lat, lateral; m, circular tufts of cilia localized to the mouth; nt, neurotroch; pt, prototroch; tt, telotroch; vent, ventral; vnc, ventral nerve cord.

**Fig. 6.**
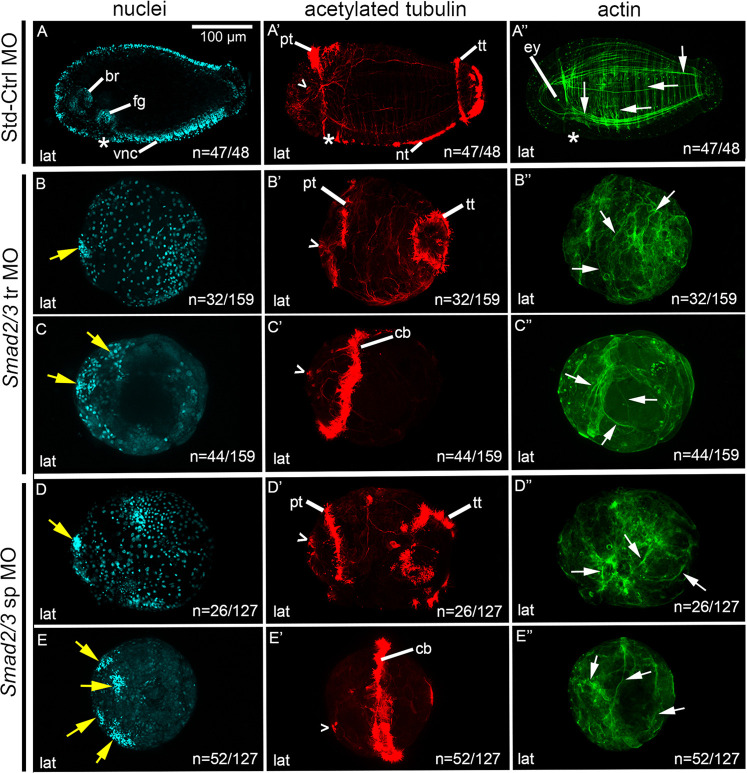
**MO knockdown of *Smad2/3* results in loss of dorsal-ventral axis.** (A-E″) Images across a row correspond to a single stage 6 larva. Larvae are laterally oriented with anterior to the left. Each panel depicts a confocal projection. Columns show labeling of nuclei with Hoechst (A-E), cilia and neurons with anti-acetylated tubulin (A′-E′) and actin filaments with phalloidin (A″-E″). (A-A″) Images of a larva resulting from zygotic injections with Std-Ctrl MO, and exhibit a wild-type-like phenotype. (B-B″,C-C″) Phenotypic series showing abnormal larvae resulting from zygotic injections of a *Smad2/3* tr MO. (D-D″,E-E″) Phenotypic series showing abnormal larvae resulting from zygotic injections of a *Smad2/3* sp MO. Open arrowheads, sc^ac+^ ; asterisks, position of the mouth; white arrows, circumferential and longitudinal muscle fibers; yellow arrows, clusters of nuclei. br, brain; cb, ciliary band; ct, ciliary tufts; ey, eye; fg, foregut; lat, lateral; nt, neurotroch; pt, prototroch; tt, telotroch; vnc, ventral nerve cord.

Stage 6 larvae possess morphological features that allow for clear identification of an anterior-posterior axis, dorsal-ventral axis and bilateral symmetry using Hoechst dye, phalloidin and an anti-acetylated tubulin antibody (Fig. 5) (Meyer et al., 2015; Seaver et al., 2005). Detectable anterior features included the brain, visible by nuclear staining (Fig. 5A,B); acetylated tubulin-positive sensory cells (sc^ac+^) (Fig. 5A′, open arrowhead); a ciliary band called the prototroch (Fig. 5A′,B′); and two larval eyes (Fig. 5A″,B″), whose microvilli in the photosensory cell are visible with F-actin staining (Yamaguchi and Seaver, 2013). A posteriorly positioned ciliary band called the telotroch was also present (Fig. 5A′,B′). Together, these characteristics indicate a well-defined anterior-posterior axis. Ventral features included the ventral nerve cord (VNC) (Fig. 5A,B′), foregut, mouth (Fig. 5A,B) and a ventral ciliary band called the neurotroch that is distinguishable by its short cilia (Fig. 5A′). One dorsal landmark was the sc^ac+^, positioned on the dorsal edge of the brain (Fig. 5A′). Together, the dorsal sc^ac+^ coupled with the aforementioned ventral features indicate a clear dorsal-ventral axis. Bilateral symmetry was detectable via the presence of two brain lobes, bilateral foregut anlagen (Fig. 5B), two eyes and the symmetrical arrangement of longitudinal and circular muscles (Fig. 5B″). All experimental and control MO injected zygotes were raised to stage 6 larvae and scored for all three body axes. Morphants resulting from microinjections of 800 µM of the Std-Ctrl MO phenotypically resembled wild-type larvae (Fig. 6A-A″), and all three body axes were clearly evident in 47/48 cases (98%; five biological replicates).

### MO knockdown of *Smad2/3* significantly decreases dorsal-ventral axis formation

Stage 6 morphant larvae resulting from microinjections of 800 µM *Smad2/3* tr MO (Fig. 6B-C″) were reproducibly abnormal in 117/159 cases and possessed wild-type-like morphology in 42/159 cases (five biological replicates). There was a phenotypic range of abnormal larvae, and in 76/117 cases, a dorsal-ventral axis and bilateral symmetry were not detectable (Fig. 6B-C″). However, these abnormal morphants possessed features indicative of anterior identity. There were several small dense clusters of nuclei localized to one end of the larva (Fig. 6B,C; yellow arrows). Localized to the same end of the larva were sc^ac+^ (Fig. 6B′,C′). The colocalization of clusters of nuclei and sc^ac+^ suggests the presence of disorganized neural tissue. Features similar to these were previously identified as neural tissue using a molecular marker for differentiating neurons, *CapI-elav1*, in larvae resulting from chemical inhibition of the Activin/Nodal pathway (Lanza and Seaver, 2018). In addition, these abnormal morphants possessed a range of ciliation patterns: some had clear anterior and posterior ciliary bands indicating anterior-posterior polarity (32/76) (Fig. 6B′) and others possessed either a medially positioned ciliary band (Fig. 6C′) or ciliary tufts that could not be identified as prototroch or telotroch (44/76), but served as a landmark against which distinguishable features could be oriented. There were few actin fibers present relative to controls, and those present were disorganized (Fig. 6B″,C″). Furthermore, these larvae lacked a visible mouth invagination, foregut tissue and ventral neurotroch. Together, these data indicate that there is anterior identity on one end of the larvae relative to the single medial ciliary band, and a clear anterior-posterior axis in those with two ciliary bands. A dorsal-ventral axis and bilateral symmetry were not detectable.

The remaining abnormal morphants (41/117) possessed features indicative of an anterior-posterior axis, a dorsal-ventral axis and bilateral symmetry (not shown). However, these morphants possessed abnormalities such as an abnormal VNC and neurotroch, and lacked a detectable mouth invagination or foregut anlage. There were also reduced numbers of circumferential actin fibers and no detectable longitudinal fibers. In summary, the *Smad2/3* tr MO resulted in 42/159 wild-type-like morphants, 41/159 abnormal morphants with a detectable dorsal-ventral axis and 76/159 abnormal morphants that lacked a detectable dorsal-ventral axis.

Similarly, morphant larvae resulting from microinjections with 800 µM *Smad2/3* sp MO (Fig. 6D-E″) were reproducibly abnormal in 112/127 cases, and possessed wild-type-like morphology in 15/127 cases (four biological replicates). In 78/112 abnormal morphants, a dorsal-ventral axis and bilateral symmetry were not distinguishable (Fig. 6D-E″). However, these morphants possessed differentiated cell types and anterior polarity. Several small dense clusters of nuclei (Fig. 6D, E) were present in conjunction with the sc^ac+^ (Fig. 6D′,E′), suggestive of disorganized neural tissue. Some morphants also possessed a prototroch, reduced telotroch and lacked a neurotroch (26/78) (Fig. 6D′). In the majority of cases (52/78), however, these morphants possessed a single medial ciliary band, which encircled the larva (Fig. 6E′) or medially positioned ciliary tufts. Morphants had few actin fibers, and they were disorganized (Fig. 6D″,E″). Because there was no indication of a VNC, mouth or foregut, dorsal-ventral and bilateral symmetry could not be detected.

The remaining 34/112 abnormal morphants possessed features indicative of anterior-posterior and dorsal-ventral axes, and bilateral symmetry (not shown). Abnormalities seen in these morphants included the lack of a VNC and foregut anlage. A reduced neurotroch was present, and there were circular F-actin fibers but few longitudinal fibers.

In summary, *Smad2/3* sp MO resulted in 15/127 cases that had a wild-type-like appearance, 34/127 abnormal morphants with a detectable dorsal-ventral axis and 78/127 abnormal morphants that lacked a detectable dorsal-ventral axis. Furthermore, both *Ct-Smad2/3* MOs generated comparable morphant phenotypes, providing confidence that the observed phenotype was specific and the result of knockdown of the target, *Smad2/3*.

**Fig. 7.**
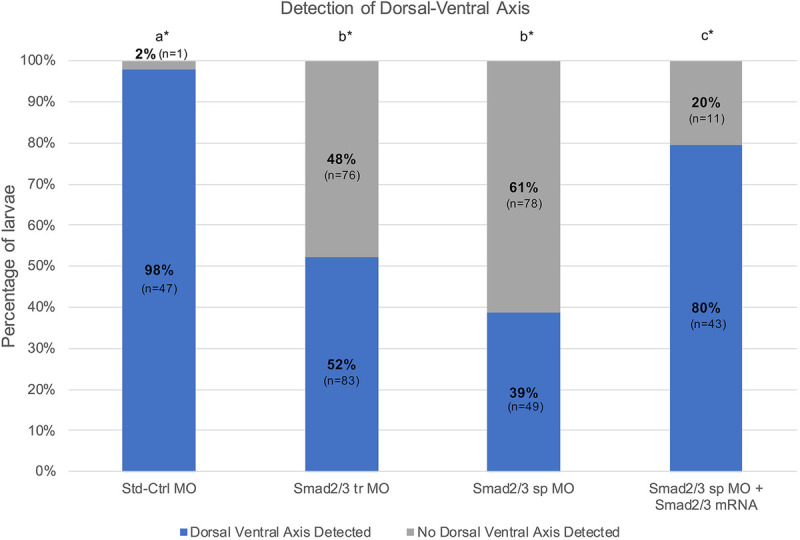
**Reduction of dorsal-ventral axis in *Smad2/3* knockdowns and rescue with *Smad2/3* mRNA.** Percentage of animals with a detectable dorsal-ventral axis (blue) in relation to the percentage of animals lacking a detectable dorsal-ventral axis (gray). There is significance between treatment conditions if the letters with asterisks differ. Overlapping letters do not differ at *P*=0.05 using post-hoc Bonferroni-corrected *z*-test of two proportions.

The proportion of larvae with a dorsal-ventral axis was compared among the Std-Ctrl, *Smad2/3* tr and *Smad2/3* sp conditions (Fig. 7). An omnibus Chi-square test of homogeneity showed at least one statistically significant difference in proportions (*P*<0.000) between conditions. *Smad2/3* tr morphants had a detectable dorsal-ventral axis in 52% (83/159) of cases. In *Smad2/3* sp morphants, a dorsal-ventral axis was detectable in 39% (49/127) of the larvae. Post-hoc pairwise comparisons using a *z*-test of two proportions revealed no statistically significant difference (*P*>0.05) between either knockdown conditions; however, both *Smad2/3* MOs resulted in a significantly lower proportion of larvae with a detectable dorsal-ventral axis compared with the Std-Ctrl condition (*P*<0.05) (Fig. 7).

### Rescue of sp MO phenotype with *Smad2/3* mRNA

**Fig. 8.**
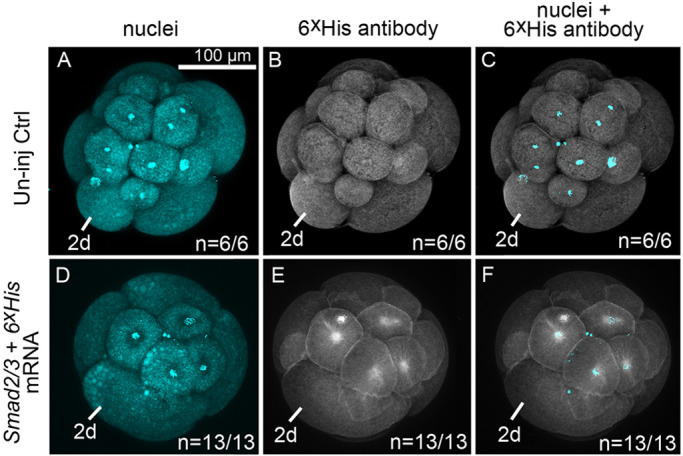
***Smad2/3* mRNA is translated into protein by the 16-cell stage.** (A-F) *Z*-stacks of merged confocal fluorescent images of 16-cell stage embryos. (A-C) A single uninjected control embryo. (D-F) A single embryo resulting from zygotic injection with 3′ 6×His-tagged mRNA. (A,D) DNA is labeled in cyan by Hoechst staining. (B,E) Embryos labeled with the anti-6×His monoclonal antibody detect recombinant protein containing the 6×His epitope tag (white in E). (C,F) Merged image of D and E showing spatial relationship between nuclear labeling and Smad2/3.

Exogenous *Smad2/3* mRNA was able to rescue *Smad2/3* sp morphant phenotypes. Recombinant Smad2/3 protein was detectable in 16-cell stage embryos following microinjection of 3′ 6×His-tagged mRNA into zygotes, demonstrating efficient translation of *Smad2/3* mRNA (Fig. 8).

**Fig. 9.**
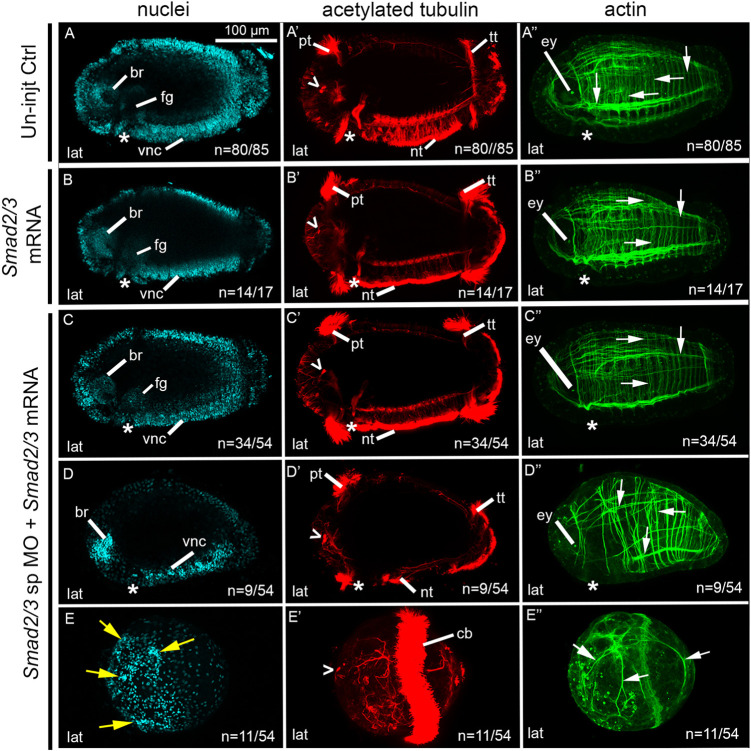
**Exogenous *Smad2/3* mRNA rescues *Smad2/3* splice-blocking morpholino phenotypes.** (A-E″) Images across a row correspond to a single stage 6 larva. Larvae are laterally oriented with anterior to the left. Each panel depicts a confocal projection. Each column depicts labeling for nuclei with Hoechst (A-E), cilia and neurons with anti-acetylated tubulin (A′-E′) or actin filaments with phalloidin (A″-E″). (A-A″) Images of larva with wild-type-like phenotype resulting from uninjected control embryos. (B-B″) Larva resulting from zygotic injections of wild-type *Smad2/3* mRNA. (C-E″) Larva resulting from zygotic injections of both *Smad2/3* sp MO and wild-type *Smad2/3* mRNA. Phenotypically, larvae are wild-type-like (C-C″), moderately abnormal (D-D″) or severely abnormal (E-E″). Open arrowheads, sc^ac+^; asterisks, position of the mouth; white arrows, muscle fibers; yellow arrows, clusters of nuclei. br, brain; cb, ciliary band; ey, eye; fg, foregut; lat, lateral; nt, neurotroch; pt, prototroch; tt, telotroch; vnc, ventral nerve cord.

In rescue experiments, zygotes were microinjected with either *Smad2/3* mRNA or a combination of *Smad2/3* sp MO plus *Smad2/3* mRNA and raised to larval stage 6 for phenotypic analysis. Uninjected embryos resulted in wild-type larvae (Fig. 9A-A″). Zygotes injected with 100 ng/μl *Smad2/3* mRNA alone resulted in mostly wild-type-like larvae (14/17) (Fig. 9B-B″), with only a small proportion of abnormal larvae (3/17; not shown). These results demonstrate that overexpression of *Smad2/3* alone does not cause a detectable phenotype. Zygotes injected with 800 µM *Smad2/3* sp MO plus 100 ng/μl *Smad2/3* mRNA resulted in reproducible larval phenotypes (three biological replicates; Fig. 9C-E″) sorted as follows: wild-type-like (34/54), moderately abnormal (9/54) and severely abnormal (11/54). All three body axes were detected in both wild-type-like (Fig. 9C-C″) and moderately abnormal larvae (Fig. 9D-D″). In the few severely abnormal larvae (11/54) (Fig. 9E-E″), anterior identity was detectable via the presence of small clusters of densely packed nuclei (yellow arrows) and sc^ac+^ (open arrowhead) on one end of the larvae. However, bilateral symmetry or dorsal-ventral polarity could not be detected.

A dorsal-ventral axis was detectable in 80% (43/54) of cases resulting from mRNA rescue experiments (*Smad2/3* sp MO plus *Smad2/3* mRNA) (Fig. 7). The proportion of larvae with a detectable dorsal-ventral axis in the rescue condition was statistically significantly higher than in animals in the *Smad2/3* sp MO and *Smad2/3* tr MO conditions (*P*<0.05), yet statistically significantly lower than animals in the control condition (*P*<0.05) (Fig. 7). Therefore, wild-type *Smad2/3* mRNA significantly rescues the *Smad2/3* sp MO phenotype.

### MO knockdown of *Smad1/5/8* results in larvae with a dorsal-ventral axis

**Fig. 10.**
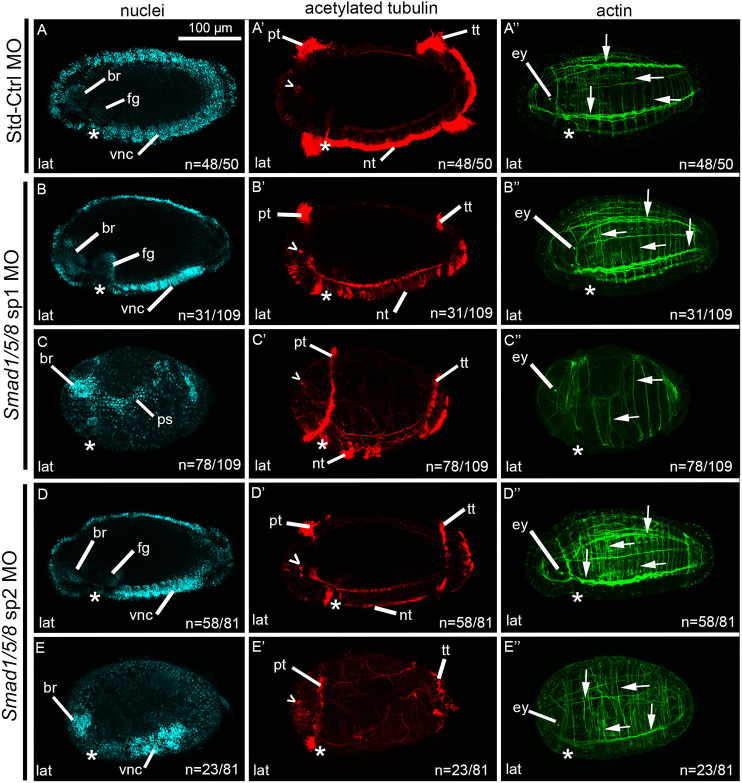
**MO knockdown of *Smad1/5/8* results in abnormal larval morphology.** (A- E″) Images across a row are of a single stage 6 larva. Larvae are laterally oriented, anterior to the left and posterior to the right. Each panel depicts a merged confocal stack. Each column depicts labeling for nuclei with Hoechst (A-E), cilia and neurons with anti-acetylated tubulin (A′-E′) or actin filaments with phalloidin (A″-E″). (A-A″) Larva resulting from zygotic injections with the Std-Ctrl MO. *Smad1/5/8* sp1 morphants exhibit wild-type-like (B-B″) or abnormal larval phenotypes (C-C″). *Smad1/5/8* sp2 morphants exhibit wild-type-like (D-D″) or abnormal larval phenotypes (E-E″). Open arrowheads, sc^ac+^; asterisks, position of the mouth; white arrows, muscle fibers. br, brain; cb, ciliary band; ey, eye; fg, foregut; lat, lateral; nt, neurotroch; ps, presumptive segments; pt, prototroch; tt, telotroch; vnc, ventral nerve cord.

Previous investigations using chemical inhibitors indicated that BMP signaling does not contribute to dorsal-ventral axis patterning in *Capitella* (Lanza and Seaver, 2018). To verify these results, *Smad1/5/8*, a downstream transduction factor in the BMP signaling pathway, was targeted using morpholinos. In all experiments, a subset of each brood was injected with 800 µM of the Std-Ctrl MO. Std-Ctrl morphants phenotypically resembled wild-type larvae (Fig. 10A-A″) and all three body axes were clearly distinguishable (48/50, 96%).

Zygotes injected with 800 µM *Smad1/5/8* sp1 MO resulted in morphant larvae with reproducible phenotypes (five biological replicates), sortable into two general morphological categories: wild-type-like (31/109) and abnormal (78/109) (Fig. 10B-C″). The 31/109 larvae resulting from *Smad1/5/8* sp1 MO injections possessed a phenotype similar to wild type with all three body axes and differentiated tissues (Fig. 10B-B″). The abnormal *Smad1/5/8* sp1 morphants had anterior features such as bilateral brain lobes (Fig. 10C), sc^ac+^ along the dorsal edge of the brain (Fig. 10C′), a prototroch (Fig. 10C′) and two eyespots (Fig. 10C″). Combined with the posteriorly occurring telotroch (Fig. 10C′), these features indicate an anterior-posterior axis. These larvae also possessed a mouth (Fig. 10C) and tufts of short cilia on one side of the trunk, which are probably neurotroch (Fig. 10C′). These ventral features in combination with the dorsally positioned sc^ac+^ indicate the presence of a dorsal-ventral axis in 59/78. These larvae lacked VNC ganglia, although most had longitudinal nerves near the ventral midline. A longitudinal band of loosely organized nuclei was present in the ventro-lateral trunk at the approximate location of the segmental precursors (Fig. 10C, ps), suggesting a defect in the segmentation process. There were a reduced number of actin fibers, most of which were circumferentially arranged, and a few longitudinal fibers (Fig. 10C″). The presence of bilateral brain lobes (Fig. 10C) and two eyes (Fig. 10C″) established bilateral symmetry. In the remaining 19 of the 78 abnormal morphants, a dorsal-ventral axis and bilateral symmetry were undetectable. These larvae had a small number of disorganized actin fibers and lacked visible indications of a mouth, foregut or neurotroch. Additionally, these larvae possessed a single central condensation of nuclei in the head that may be indicative of neural tissue. This feature in combination with the anterior and posterior ciliary bands suggests the presence of an anterior-posterior axis. However, no other axes were detectable. In summary, microinjection of *Smad1/5/8* sp1 MO results in 31/109 wild-type-like larvae and 59/109 abnormal morphants with a detectable dorsal-ventral axis. Only 19/109 morphants lack a detectable dorsal-ventral axis.

Similarly, injections with 800 µM of the second splice-blocking MO targeting *Smad1/5/8* resulted in most larvae possessing all three body axes (Fig. 10D-E″; four biological replicates). Morphants were categorized by general morphology as either wild-type-like (58/81; Fig. 10D-D″) or abnormal (23/81; Fig. 10E-E″). In 20 of the 23 abnormal morphants, an anterior-posterior axis was identifiable via the anteriorly positioned brain (Fig. 10E), sc^ac+^ (Fig. 10E′), prototroch (Fig. 10E′), eyes (Fig. 10E″) and a posterior telotroch (Fig. 10E′). A dorsal-ventral axis was also identifiable in 20/23 cases by the presence of a mouth, VNC (Fig. 10E) and the sc^ac+^ (Fig. 10E′). A subset of these morphants (13/23) had scattered tufts of short cilia, indicative of a reduced neurotroch, located on the same face of the larval trunk as the mouth. In 7/23 morphants, a normal neurotroch was present. There were both circular and longitudinal actin fibers, although fewer than in wild-type larvae (Fig. 10E″). Lastly, the presence of bilateral brain lobes (Fig. 10E) and two eyes (Fig. 10E″) established bilateral symmetry (20/23). In 3/23 of the abnormal morphants, a dorsal-ventral axis was not detectable. In summary*,* microinjection of *Smad1/5/8* sp2 MO results in 58/81 wild-type-like larvae, 20/81 phenotypically abnormal morphants with a detectable dorsal-ventral axis and only 3/81 abnormal morphants with no detectable dorsal-ventral axis.

**Fig. 11.**
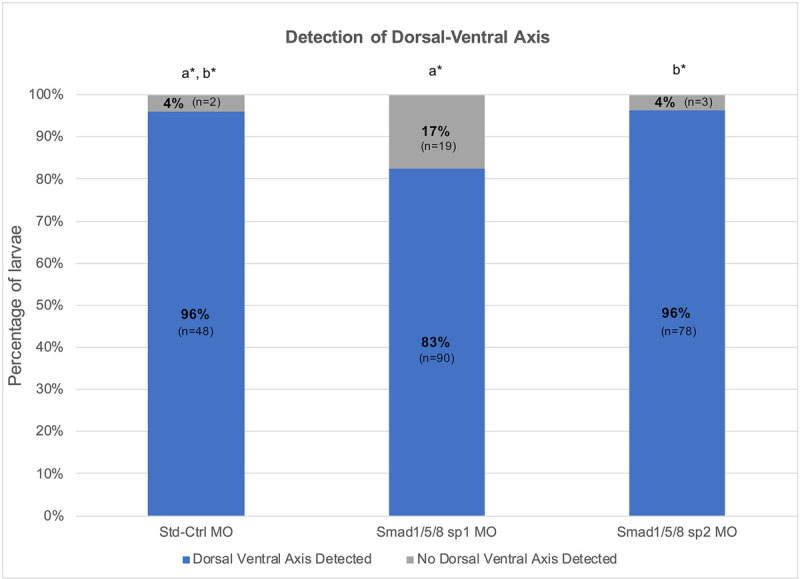
**Detection of dorsal-ventral axis in *Smad1/5/8* knockdowns.** Percentage of animals in which dorsal-ventral axis is detectable (blue) in relation to the percentage of animals in which a dorsal-ventral axis is not detectable (gray). There is significance between treatment conditions if the letters with asterisks differ. Overlapping letters do not differ at *P*=0.05 using post-hoc Bonferroni-corrected *z*-test of two proportions.

The proportion of larvae with a dorsal-ventral axis was compared among the Std-Ctrl, *Smad1/5/8* sp1 and *Smad1/5/8* sp2 conditions (Fig. 11). An omnibus Chi-square test of homogeneity showed at least one statistically significant difference in proportions (*P*<0.05) between conditions. A dorsal-ventral axis was detectable in 83% (90/109) of *Smad1/5/8* sp1 morphant larvae, 96% (78/81) of *Smad1/5/8* sp2 morphant larvae and 96% (48/50) of cases for Std-Ctrl larvae. Post-hoc pairwise comparisons using a *z*-test of two proportions revealed a statistically significant difference between the two *Smad1/5/8* MO conditions (*P*<0.05); however, there was no significant difference in the proportion of larvae with a detectable dorsal-ventral axis when either of the *Smad1/5/8* MO conditions was compared with the Std-Ctrl condition (*P*>0.05) (Fig. 11). Because neither *Smad1/5/8* MO significantly affected dorsal-ventral patterning, we conclude that *Smad1/5/8* does not have a primary role in patterning the dorsal-ventral axis during organizing activity.

## DISCUSSION

### Activin/Nodal branch of the TGF-β superfamily is the primary signaling pathway functioning in *Capitella* dorsal-ventral axis formation

In *Capitella* embryos, two different morpholinos were used to specifically target *Smad2/3*, a receptor signal transducer specific to the Activin/Nodal pathway. Knockdown of *Ct-Smad2/3* with either morpholino resulted in a statistically significant loss of the dorsal-ventral axis as compared with morphants injected with the Std-Ctrl MO (*P*<0.05). Zygotic injections with wild-type *Smad2/3* mRNA in combination with the *Smad2/3* sp MO rescued formation of the dorsal-ventral axis in 40% of cases. These data demonstrate that our MOs specifically target *Smad2/3,* and confirm that the Activin/Nodal pathway is essential for dorsal-ventral patterning.

Furthermore, these results show phenotypic similarities to larvae resulting from 2d ablations (Amiel et al., 2013). When the organizer cell is ablated, the resulting larvae are morphologically spherical, possess a single ciliary band, have radialized features in the head, and reduced specification of trunk identity. Because descendants of cell 2d contribute to the formation of trunk ectoderm, the spherical morphology and lack of identifiable trunk features is not surprising in 2d-ablated animals (Amiel et al., 2013). Further analyses of 2d-ablated animals examined tissue differentiation and axis specification using the markers *CapI-elav1,* a marker of differentiating neurons (Meyer and Seaver, 2009), and *CapI-gataB3*, a trunk marker expressed in lateral mesoderm (Boyle and Seaver, 2008). *CapI-elav1* is expressed in a radialized pattern in the head, but is not detectable in the presumptive trunk, whereas *CapI-gataB3* is expressed in a reduced domain in the posterior-most part of the trunk (Amiel et al., 2013). These data indicate a reduction in the differentiation of neural tissue and of mesodermal structures in the larval trunk. Our *Smad2/3* MO morphants possess a similar but less severe phenotype. For instance, *Smad2/3* morphants have radialized head features, but a greater proportion of morphants have structures indicative of trunk identity despite lacking dorsal-ventral patterning. In Activin/Nodal-inhibited animals, we previously demonstrated that 2d descendants contribute to the presumptive trunk in larvae (Lanza and Seaver, 2018). Therefore, the differences between 2d ablation and *Smad2/3* MO phenotypes probably arise because the 2d lineage persists in *Smad2/3* morphants.

We also targeted *Smad1/5/8*, a receptor signal transducer specific to the BMP pathway, using two different splice-blocking morpholinos. A statistically significant proportion of morphants resulting from microinjection of either *Smad1/5/8* MO possess a clear dorsal-ventral axis. Signaling via *Ct-Smad1/5/8* is therefore not the primary dorsal-ventral patterning signal. Detailed analysis is required to fully understand the role of BMP in *C. teleta* development; however, the phenotype we observe hints at a role in central nervous system development, gangliogenesis and muscle fiber organization.

These data support previous findings that the BMP signaling pathway is not essential in dorsal-ventral axis patterning in *C. teleta* (Corbet, 2016; Lanza and Seaver, 2018). Specifically, Lanza and Seaver (2018) reported that BMP signaling is inhibited by using the chemical inhibitor dorsomorphin dihydrochloride during the time interval associated with organizer activity. Although drug exposure resulted in abnormal larval phenotypes, a dorsal-ventral axis was identifiable, suggesting that the primary organizing activity signal is not mediated by BMP signaling (Lanza and Seaver, 2018). The MO knockdown data in this study suggest that although BMP signaling functions in early *C. teleta* development, it does not have a primary role in dorsal-ventral axis patterning. To better understand the mechanism(s) of dorsal-ventral patterning in Annelida, future investigations should seek to sample additional annelid clades (Weigert and Bleidorn, 2016).

### Evolution of dorsal-ventral patterning

Our findings highlight taxonomic differences in the molecular mechanisms of axis patterning among members of Spiralia and within Bilateria as a whole. In some Spiralia, dorsal-ventral patterning occurs via BMP signaling (Clement, 1952; Kuo and Weisblat, 2011; Lambert et al., 2016; Tan et al., 2017, 2018). This has been demonstrated in two mollusks, the gastropod *Tritia obsoleta* (formerly *Ilyanassa obsoleta*) and the bivalve *Crassostrea gigas* (Lambert et al., 2016; Tan et al., 2018). In *T. obsoleta,* MO knockdown of *loDpp* prevents the development of larval structures that require an inductive organizer signal such as eyes and external shell (Lambert et al., 2016). A similar result is seen following ablation of the polar lobe in *T. obsoleta*. The polar lobe is a transient protrusion that forms during the first few cell divisions in spiralians, contains cytoplasmic determinants that are specifically shunted into the CD and D blastomeres, and functions in specifying the organizer cell (Clement, 1952). Following *T. obsoleta* polar lobe deletion, dorsal-ventral identity can be restored by addition of exogenous BMP4, which induces the formation of organizer-dependent structures (Lambert et al., 2016). Likewise, in *C. gigas,* embryonic inhibition of BMP signaling with dorsomorphin dihydrochloride affects dorsal-ventral gene expression at the gastrula stage (Tan et al., 2018).

BMP signaling is also linked to dorsal-ventral patterning in other spiralians. Although brachiopods do not develop according to a stereotyped spiral cleavage program (Martín-Durán et al., 2016), the expression of ventral ectodermal markers expand dorsally following continuous exposure to the BMP inhibitor DMH1 at the two-cell to early larval stage in *Novocrania anomala* and *Terebratalia transversa*. Similarly, in the annelid *Helobdella robusta,* MO knockdown of *BMP5-8* negatively affects dorsal patterning, whereas knockdown of *gremlin*, an extracellular BMP ligand inhibitor, revealed that ventral patterning relies on the inhibition of other broadly expressed BMPs (Kuo and Weisblat, 2011). Notably, *H. robusta* embryos develop via a modified spiral cleavage program (Weisblat, 1999, 2007), perhaps contributing to molecular differences in axial patterning with *C. teleta*.

In addition to Spiralia, dorsal-ventral axis patterning in embryos requires BMP signaling in numerous deuterostomes and ecdysozoans. In arthropods such as *Drosophila melanogaster* (Eldar et al., 2002; Lynch and Roth, 2011), chordates such as *Xenopus laevis,* (De Robertis and Kuroda, 2004) and hemichordates such as *Saccoglossus kowalevskii* (Lowe et al., 2006), BMP signaling is required for axis patterning. Interestingly, although BMPs do function in patterning sea urchin embryos, it is Nodal, an Activin/Nodal family ligand, that is required to initiate the downstream expression of *BMP2/4* (Duboc et al., 2004; Lapraz et al., 2015). Furthermore, ectopic expression of *nodal* in sea urchin embryos is sufficient to induce the formation of a second dorsal-ventral axis (Duboc et al., 2004; Lapraz et al., 2015). Few studies have specifically investigated the role of Activin/Nodal signaling in dorsal-ventral patterning. One example comes from the early branching annelid *Chaetopterus pergamentaceus,* in which the inhibition of Activin/Nodal signaling with SB431542 during early cleavages prevents dorsal-ventral patterning (Lanza and Seaver, 2020). It is possible that additional species utilize both branches of the TGF-β signaling cassette in dorsal-ventral patterning. However, in the gastropods *Biomphalaria glabrata* and *Lottia gigantea* this is not the case, as the inhibition of Nodal by the inhibitor SB431542 during early embryogenesis affects left-right but not dorsal-ventral patterning (Grande and Patel, 2009).

Studies in some animals have suggested that dorsal-ventral patterning does not occur via BMP signaling. For example, dorsal-ventral axis formation is not disrupted by mutant BMP-like pathway genes in *C. elegans*, making it unlikely that BMP signaling functions in this patterning event (Patterson and Padgett, 2000). Similarly, a recent investigation in the slipper snail *Crepidula fornicata* demonstrated that BMP signaling does not play a primary role in organizer function (Lyons et al., 2020). In *C. fornicata*, inhibition of BMP signaling by embryonic exposure to DMH1 results in larvae with head defects and a detectable dorsal-ventral axis in the trunk (Lyons et al., 2020). Addition of ectopic BMP4 protein also results in larvae with a detectable dorsal-ventral axis (Lyons et al., 2020). The data presented in our study provides another novel instance in which BMP signaling is demonstratively not the primary pathway involved in embryonic dorsal-ventral axis patterning. Instead, we provide strong evidence that Activin/Nodal signaling functions as the primary dorsal-ventral patterning pathway in the annelid *C. teleta*.

Our results may provide an example of developmental systems drift, whereby the underlying developmental mechanisms involved in the formation of homologous features differ between related species (True and Haag, 2001). A classic example comes from a comparison of development between the nematodes *C. elegans* and *Pristionchus pacificus*. In both species, the vulva is derived from homologous precursor cells. However, vulval induction occurs via EGF signaling in *C. elegans*, but via Wnt signaling in *P. pacificus* (Sommer, 2012; Sternberg, 2005; True and Haag, 2001; Wang and Sommer, 2011). Another example comes from *Ciona intestinalis* and *Molgula occidentalis*, two ascidians with very similar cleavage programs and whose blastomeres share similar cell fates, yet with clear differences in the *cis-*regulatory sequences controlling cardiopharyangeal development (Stolfi et al., 2014).

In spiralians, embryonic development follows a highly conserved, stereotypic cleavage program that allows for comparison of homologous cells across taxa. Structures such as the eyes, trunk mesoderm and trunk ectoderm are generally derived from homologous embryonic precursors (Ackermann et al., 2005; Hejnol, 2010; Meyer et al., 2010). In many embryos examined, the D quadrant has the ability to establish dorsal-ventral polarity (Dorresteijn et al., 1987; Hejnol, 2010; Henry and Martindale, 1987; Render, 1983, 1989) and gives rise to the spiralian organizer cell (Amiel et al., 2013; Clement, 1962; Damen and Dictus, 1996b; Henry and Perry, 2008; Henry et al., 2006; Lambert and Nagy, 2003). The genomes of annelids and mollusks have very similar components of both branches of the TGF-β signaling cassette (Kenny et al., 2014). Therefore, differences in gene content do not explain the molecular differences in dorsal-ventral patterning. Ultimately, differences in the molecular identity of the dorsal-ventral patterning signal, in spite of having a shared early embryonic developmental program and shared D quadrant role in dorsal-ventral axis specification, might be an example of the developmental systems drift that occurred within Spiralia over evolutionary time.

### Proposed model for organizing activity in *C. teleta*

**Fig. 12.**
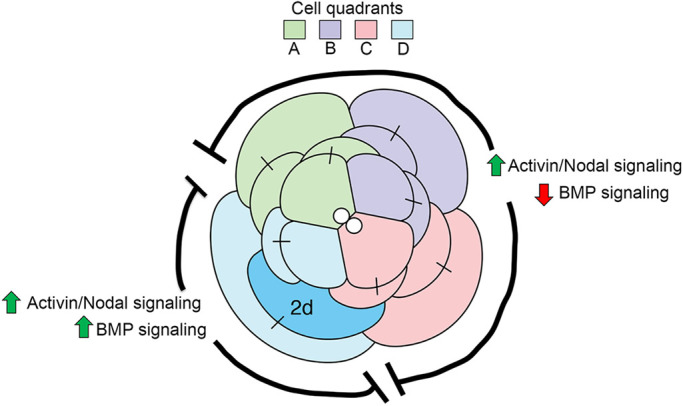
**Hypothesized model for organizing activity.** Schematic depicting 16-cell stage embryo during organizing activity by micromere 2d. Identity of each cell quadrant is specified via the color key. Model suggests BMP signaling is downregulated (red arrow) in quadrants A, B and C but not in D (green arrow), whereas Activin/Nodal signaling is active in all four quadrants (green arrows).

Our MO knockdown data indicate a primary role for Activin/Nodal signaling during embryonic dorsal-ventral patterning. Given our expression data and these MO results, we hypothesize that the mechanism by which the TGF-β superfamily operates during organizing activity involves the downregulation of BMP signaling via action of inhibitors to allow for the preferential activity of Activin/Nodal signaling (Fig. 12). For both the Activin/Nodal and BMP pathways, the ligands, receptors and signal transducers are expressed in all four embryonic quadrants when 2d is present. This is evidenced by the expression of *Activin/inhibin/myostatin-like 5*, *BMP Receptor 2* and *Smad1/5/8* in first quartet cells, and by our previous study that detected two Activin/Nodal receptors and *Smad2/3* in all quadrants of the first and second quartet cells (Lanza and Seaver, 2018). In contrast, the spatial restriction of the BMP modulators *TSG* and *Noggin A* suggests a model in which BMP signaling is downregulated in quadrants A, B and C of the embryo, but not in the D quadrant (Fig. 12).

Both TSG and Noggin A antagonize BMP signaling by directly binding to BMP ligands and preventing receptor activation (Blitz, 2003; Chang et al., 2001; Krause et al., 2011; Zimmerman et al., 1996). Notably, in some species, TSG also promotes BMP signaling by cleaving Chordin-bound BMPs (Oelgeschläger et al., 2000). Annelids, including *Capitella,* appear to have lost *Chordin*, but possess a similar gene, *Chordin-like* (Kenny et al., 2014). Although it is possible that TSG and Chordin-like proteins function similarly, the relative expression levels of *Chordin-like* are very low at the time points examined.

In *Xenopus*, the organizer secretes several inhibitory molecules, including Noggin, to prevent ectoderm from being induced into neural tissue (Sasai, 1994; Smith and Harland, 1992; Smith et al., 1993). Our model proposes a similar mechanism whereby inhibitory molecules provide some specificity during organizer signaling. Future functional investigations that target *TSG* and *Noggin A* would provide support for this model.

The identity of the ligand that initiates organizer activity in *Capitella* is still unknown. In previous blastomere deletion and isolation studies, it was demonstrated that the first quartet micromeres (1a-1d) are not required for formation of a dorsal-ventral axis, and that they instead rely on an external inductive signal for dorsal-ventral patterning of the head (Carrillo-Baltodano and Meyer, 2017; Yamaguchi et al., 2016). This suggests that ligands expressed by the first quartet blastomeres do not have a role in dorsal-ventral specification. Future investigations should examine expression patterns of the remaining Activin/Nodal ligands.

### Modification of Smad domain structure

An unexpected finding from this study is the identification of a modification in the domain structure of the *Smad1/5/8* gene. In general, Smads contain two conserved protein domains: an MH1 domain (Mad homology domain 1) towards the N terminus and an MH2 domain (Mad homology domain 2) towards the C terminus (Patterson and Padgett, 2000; Zavala-Góngora et al., 2008). The MH1 domain functions in DNA binding and the MH2 domain mediates protein-protein interactions via direct binding to the intracellular portion of transmembrane receptors. *Capitella Smad1/5/8* possesses an MH2 domain but lacks a MH1 domain. There is precedence for Smads to function with only an MH2 domain. In *C. elegans*, the protein Daf-14 functions as an inhibitory Smad, and it only possesses a well-conserved MH2 (Inoue and Thomas, 2000). Studies examining the distinct roles of the MH1 and MH2 domains in *Xenopus laevis* found that truncated Smads with only the MH2 domain can activate transcription; however, wild-type specificity is only activated in the presence of an MH1 domain (Fortuno et al., 2001). It is possible that *Ct-Smad1/5/8* has a dominant-negative function and can bind to the transmembrane receptor but not bind directly to DNA. It will be interesting to determine whether there is a similar modification of the domain structure of *Smad1/5/8* in other annelids.

### Conclusions and future directions

The results of this study reveal that in the annelid *C. teleta,* signaling via Smad2/3 of the Activin/Nodal pathway is essential for dorsal-ventral axis patterning during organizer activity. The identity of the activating ligand is still unknown. In future investigations, the spatial distribution of the Activin/Nodal ligand *Activin/inhibin/myostatin-like 1* can be determined via FISH or, ideally, protein products of all three transcribed Activin/Nodal ligands can be localized with ligand-specific antibodies in 16-cell stage embryos. Additionally, these Activin/Nodal ligands can be systematically knocked down to assess their effect on dorsal-ventral axis patterning. Given that a significant proportion of *Smad2/3 tr* MO morphants lose dorsal-ventral patterning, it is likely that the mechanism of organizing activity relies on either zygotic transcription or on maternally loaded transcript, but not on maternal protein.

The results of this study provide strong evidence that BMP signaling is not the primary inducer of dorsal-ventral axis formation in *Capitella*. Furthermore, based on expression data, we propose that BMP signaling is downregulated during organizer activity to allow for the preferential activity of Activin/Nodal signaling. Future functional investigations could confirm this. Together, our findings, along with those from the annelid *C. pergamentaceus* and the mollusk *C. fornicata* (Lyons et al., 2020), highlight important molecular differences in the induction of axes across annelids and mollusks. The extent to which Activin/Nodal signaling is essential for dorsal-ventral axis patterning in other spiralians has yet to be determined pending further sampling within this taxon.

## MATERIALS AND METHODS

### Animal care and embryo acquisition

*Capitella* adults were maintained in a laboratory colony as previously described (Grassle and Grassle, 1976; Seaver et al., 2005). Zygotes were obtained by mating reproductive male and female worms. Gravid females and sexually mature males were isolated in groups of three for 2-3 days beforehand. Before mating, animals were exposed to ambient light for approximately 6 h to create a light-stress environment. Following light exposure, mating was initiated by combining males and females into one bowl. Mating bowls were kept in the dark at room temperature for 5 h before checking for the presence of fertilized eggs.

Before fixation for *in situ* hybridization, embryos were visually monitored for birth of the organizer cell, 2d. Individual blastomeres are uniquely identifiable in *Capitella* and are named following a standard scheme used for spiralians. Following the first two cleavage divisions, the four blastomeres are named A, B, C and D, and their descendants subsequently define the four quadrants of the embryo. A, B, C and D blastomeres, known as macromeres, divide to produce multiple sets of quartets of smaller daughter cells that are called micromeres. The D quadrant macromere is usually the first to divide and, as a result, micromere 2d is always the first 2nd quartet cell to be born. However, the relative timing of the birth of 2d with respect to the division of the first quartet cells can vary by brood.

### RNA-seq developmental time course

Total RNA was extracted from individual embryos at the 8-cell (*n*=4), 16-cell (*n*=3) and 32-cell (*n*=5) stages at a fifth of the recommended volume using TRIzol (Invitrogen). Linear polyacrylamide and tRNA were added to help precipitate and visualize pellets, as well as 1 μl of the External RNA Control Consortium spike-in kit (Baker et al., 2005) at 1:500,000 dilution to help quantify amplified RNA. The TRIzol mix was added to each sample, and then samples were frozen in liquid nitrogen and thawed in a 42°C water bath five times immediately after adding TRIzol to ensure disruption of the egg membrane. Isolated RNA was eluted in ultrapure water, and a uniquely barcoded primer was added for reverse transcription and amplified according to the CEL-Seq protocol (Hashimshony et al., 2012, 2016). Samples were then sequenced on the Illumina HISeq2000 at the Technion Genome Center. CEL-Seq data was then processed as previously described using the CEL-Seq pipeline (https://github.com/yanailab/CEL-Seq-pipeline commit version fecc97e) (Hashimshony et al., 2012, 2016). Briefly, raw paired-end sequencing files were demultiplexed based on CEL-Seq barcodes into single-end fastq files. Reads were mapped against the *C. teleta* genome (GCA_000328365.1) using Bowtie2 to map the reads of the different samples in parallel. An htseq-count script was used to generate UMIs and binomial statistics were used to convert UMIs into transcript counts. The unnormalized count data generated by this pipeline was then analyzed for differential expression using a likelihood ratio test (LRT) in DESeq2 (version 1.29.5) (Love et al., 2014). This sequence data set can be accessed from GEO under accession number GSE154251. Heatmaps of TGF-β components (Table S2) were generated with normalized counts data and differentially expressed genes notated with an asterisk.

### Gene cloning

PCR was used to isolate fragments of the following genes using the following primers: *Ct-BMPR2* (NCBI accession number: ELU02740) with 5′-CAACTTACCCCACATGACGC-3′ as the forward primer and 5′- CGATGAGTGGCTGGAGTACC-3′ as the reverse primer; *Ct-Smad1/5/8* (NCBI accession number: ELU14056) with 5′-TCCTTCTCAGCCACCCAGTA-3′ as the forward primer and 5′-TGTACCATAGGCCGAGCCTA-3′ as the reverse primer; *Ct-Activin/inhibin/myostatin-like 4* (NCBI accession number: ELU11984) with 5′-TCCGCTTGGTTTCCACAACG-3′ as the forward primer and 5′-CACAGTTTACTCGATTTGTGTCCG-3′ as the reverse primer; *Ct-Activin/inhibin/myostatin-like 5* (NCBI accession number: ELT97499) with 5′-GTGGTGTCCTGCTCCTACTG-3′ as the forward primer and 5′-TAACGACACGCACACTCGT-3′ as the reverse primer; *Ct-*TSG (NCBI accession number: ELU17863) with 5′-GACGCCATCATCACGGTTAC-3′ as the forward primer and 5′-ACACGAAATTGCTCGCACAC-3′ as the reverse primer; *Ct-Noggin A* (NCBI accession number: ELU01643) with 5′- TAGCCTCGCTTCTGGTTTCA-3′ as the forward primer and 5′- ACCAAAGATGCAGGATGCAC-3′ as the reverse primer. Amplified fragments were 828 bp (*Ct-BMPR2*), 907 bp (*Ct-Smad1/5/8*), 814 bp (*Ct-Activin/inhibin/myostatin-like 5*), 1182 bp (*Ct-Activin/inhibin/myostatin-like 4*), 1017 bp (*Ct-TSG*) and 1020 bp (*Ct-Noggin A*). These were subcloned into pGEM-T Easy vector (Promega) and sequenced at the University of Hawaii or Macrogen Corp.

### Fluorescent *in situ* hybridization

Fluorescent *in situ* hybridization (FISH) was performed following previously published protocols for *C. teleta* (Klann and Seaver, 2019). Fixation of embryos entailed a 3 min membrane softening treatment consisting of exposure to a freshly made 1:1 solution of 1 M sucrose and 0.25 M sodium citrate prior to fixation with 3.7% paraformaldehyde (PFA) in filtered sea water (FSW) at 4°C overnight. Following fixation, animals were washed three times in phosphate-buffered saline (PBS) containing 0.1% Triton X-100 (PBT), twice in distilled water and then dehydrated in a series of increasing concentrations of methanol in milliQ water (25, 50, 75 and 100%). Animals were stored in 100% methanol at −20°C for a minimum of 12 h before use. Embryos were hybridized for 48 h at 65°C with each probe. Digoxigenin-labeled riboprobes for all genes were generated with either the SP6 or T7 Megascript kit (Cat No. AM1330/AM1334; Ambion). The following probes were used at a concentration of 2 ng/μl: *Ct-BMPR2*, 828 bp*; Ct-Smad1/5/8*, 907 bp; *Ct-Activin/inhibin/myostatin-like 5,* 814 bp; *Ct-Activin/inhibin/myostatin-like 4*, 1182 bp*; Ct-TSG*, 1017 bp; and *Ct-Noggin A,* 1020 bp. Detection of the digoxigenin-labeled RNA probe was carried out via a fluorescent protocol. Samples were incubated overnight with anti-digoxigenin-peroxidase (1:500), then washed eight times for 10 min in a tyramide buffer (2 M NaCl, 0.1 M boric acid, pH 8.5), followed by a 10 min incubation with a tyramide development solution (tyramide buffer, 1:1000 iodophenolboronic acid 20 mg/ml stock diluted in dimethylformamide, 1:1000 3% H_2_0_2_) plus 1:1000 rhodamine-conjugated tyramide (Hopman et al., 1998). Development was terminated by four 20 min washes with PBT, and then embryos were washed for an additional 5 days in PBT at 4°C to facilitate removal of background fluorescence. Expression occurred in slightly varying patterns across embryos (Table S1), but this correlated with the timing of the cell cycle at the point of fixation.

### Gene knockdown with morpholino oligonucleotides

Translation and splice-blocking morpholinos were designed by Gene Tools, LLC, reconstituted in nuclease-free water to a final concentration of 1 mM, and stored as directed by the manufacturer at 25°C in a dark humid environment. Prior to microinjection, MOs were diluted as described below. *Ct-Smad2/3* (NCBI accession number: EY550106) was targeted using a translation-blocking MO (5′-ACGTCATCACAAACAGATACAAGCA-3′) directed against the *Ct-Smad2/3* start site, and was expected to affect both zygotic and maternally provided *Smad2/3* mRNA. A splice-blocking MO (5′-AGTGACCTGAATGAACAGAAGCTAT-3′) binds to the boundary between intron 1 and exon 2 (*Smad2/3* sp MO).

*Ct-Smad1/5/8* (NCBI accession number: EY586111) was targeted using two non-overlapping splice-blocking morpholinos. The first MO (*Smad1/5/8* sp1 MO, 5′-CACGCATTATGTGCAGCTTACCAGG-3′) was targeted to the boundary between exon 3 and intron 3 of *Ct-Smad1/5/8*, and the second splice-blocking MO (*Smad1/5/8* sp2 MO, 5′-GTTGAAGATCTGAACACAGGACATG-3′) was directed against the boundary between intron 4 and exon 5 of *Ct-Smad1/5/8*. PCR amplification of *Ct-Smad1/5/8* identified an ATG start site immediately 5′ of the MH2 domain. Following additional amplification further 5′ of this ATG site, we were unable to identify an additional ATG start site or sequences resembling an MH1 domain. A generic standard control MO (5′-CCTCTTACCTCAGTTACAATTTATA-3′; Gene Tools) was used to control for nonspecific morpholino-induced toxicity in all experiments. Sequences of all MOs used in this study were compared with the *C. teleta* genome (Simakov et al., 2012) and confirmed to have no predicted off-target binding sites.

To confirm splice blocking activity, the targeted site of each splice-blocking MO was amplified using PCR. Resulting band sizes were expected to include the targeted intron and therefore be larger than wild-type bands. Agarose gel percentages were optimized for the size of ladders used. A 1% gel was used with a 1 kb DNA ladder, 3% gel with a 50 bp DNA ladder and 1.5% gel with a 100 bp DNA ladder.

### cDNA and mRNA synthesis

To confirm splice blocking of the MOs, RNA was extracted from pooled morphant larvae and used to synthesize cDNA (TRIzol Plus RNA Purification Kit, Thermo Fisher Scientific, Cat. No. 12183555; Super Script III First-Strand Synthesis System, Thermo Fisher Scientific, Cat No. 18080051). Typically, we used 400 ng of RNA for each cDNA synthesis reaction (total volume 20 µl). cDNA was generated in the same manner from pooled larvae resulting from microinjection of the Std-Ctrl MO. Synthesized cDNA was used in a reverse-transcription polymerase chain reaction (RT-PCR) reaction using primers designed to amplify the region surrounding the MO-targeted exon-intron boundary sequences. The *Smad2/3* sp MO target region was amplified using forward primer 5′-AAATCCATCTCCACTCAGGACC-3′ and reverse primer 5′-GTGAGTAGTGGTAGGGGTTGATAC-3′. The *Smad1/5/8* sp1 MO target region was amplified using forward primer 5′-CAGCTATCCCCATGGATTCCC-3′ and reverse primer 5′-AGGATGACCATGACTGCTCG-3′. The *Smad1/5/8* sp2 MO target region was amplified using forward primer 5′-GTCACGAGGAAAGCGTGTAGAT-3′ and reverse primer 5′-GCCTCTCCCACACGGTTGTT-3′. Std-Ctrl morphant cDNA was used as an accompanying control in all RT-PCR reactions. RT-PCR fragments were separated by gel electrophoresis. One biological replicate consisted of approximately 100-200 pooled stage 6 morphant larvae. Once larvae were placed in TRIzol, samples were stored at −80°C until further processing.

The complete *Ct-Smad2/3* coding sequence was amplified by RT-PCR from mixed larval stage cDNA using forward primer 5′-ATGACGTCGATGCTAGCGCCTTTTAC-3′ and reverse primer 5′-CTACGACATGGACGAACAGGGCATAC-3′. A *Ct-Smad2/3* mRNA with a 6×His tag on the 3′ end was also generated (Kanca et al., 2017). For this, the forward primer 5′-ATGACGTCGATGCTAGCGCCTTTTAC-3′ was used along with the reverse primer 5′-CTAATGATGATGATGATGATGCGACATGGACGAACAGGGCATAC-3′ in a PCR reaction. The amplicon was gel-purified, cloned into the pGEM-T Easy vector (Promega), and sequenced at Macrogen Corp. *Ct-Smad2/3* was then PCR-amplified from the plasmid vector using standard SP6 and T7 primers, gel-purified and used as template for an *in vitro* transcription reaction using the Megascript SP6/T7 transcription kit (Cat No. AM1330/AM1334; Ambion). A poly (A) tail was then added to the 3′ end using an Ambion Poly (A) Tailing Kit (Cat No. AM1350, Thermo Fisher Scientific). Purified mRNA was solubilized in nuclease-free water and then diluted to a stock concentration of 800 ng/μl. *Ct-Smad2/3* mRNA was microinjected either alone or with *Smad2/3* sp MO into fertilized eggs.

### Microinjections

Zygotes were prepared for microinjections with a membrane softening pretreatment. Embryos were exposed to a freshly made 1:1 solution of 1 M sucrose and 0.25 M sodium citrate for 20 s followed by three quick rinses with FSW (Meyer et al., 2010). Individual zygotes were pressure injected with quartz glass needles (QF100-70-10; Sutter Instruments) that were pulled using a micropipette puller (*P*-2000; Sutter Instruments). Needles were filled with a cocktail of 800 μM MO antisense oligonucleotides, nuclease-free water and a 1:10 dilution of 20 mg/ml red dextran reconstituted in FSW (Texas Red, Molecular Probes). The volume of microinjected MO cocktail was typically between 0.5 and 2% of the total embryo volume, as estimated from injection into an oil drop, and resulted in a final MO concentration of 4-12 µM. For rescue experiments, needles were filled with 100 ng/μl of *Ct-Smad2/3* mRNA plus dextran, 100 ng/μl *Ct-Smad2/3* mRNA 3′ 6×His tag plus dextran, or a combination of 100 ng/μl *Ct-Smad2/3* mRNA with 800 μM MO plus dextran. Injected and uninjected animals from the same brood were raised in FSW containing 60 μg/ml penicillin and 50 μg/ml streptomycin in separate dishes, and compared to assess overall brood health. A brood was considered healthy if more than 90% of the uninjected animals developed normally.

### Immunolabeling and phenotypic analysis

Larvae were fixed according to a published protocol (Lanza and Seaver, 2018). Larvae were relaxed for 10 min in a 1:1 dilution of 0.37 M MgCl_2_ in FSW followed by fixation with 3.7% PFA in FSW for 30 min. Fixative was removed and animals rinsed twice with PBS. Animals were either stored in PBS at 4°C for up to 1 month or used immediately for immunolabeling and phalloidin staining. For embryos, a 3 min membrane softening treatment consisting of a 1:1 solution of 1 M sucrose and 0.25 M sodium citrate was used before fixation with 3.7% PFA in FSW for 30 min.

Fixed animals or post *in situ* hybridization embryos were washed twice over 5 min with PBT followed by a 1 h exposure at room temperature to a blocking solution of PBT containing 10% heat-inactivated goat serum (Cat No. G9023; Sigma-Aldrich). The primary antibodies used were a 1:400 dilution of mouse anti-acetylated tubulin in blocking solution (Cat No. T6793, Lot No. 017M4806 V; Sigma-Aldrich) for larval samples, a 1:50 dilution of a 6×His tag antibody conjugated to Alexa Fluor 488 (Cat. No. MA1135A488, Lot No. UA276373; Thermo Fisher Scientific) in blocking solution for embryos and a 1:500 dilution of mouse anti-histone (Cat No. MABE71, Lot no. JC1690769; Millipore) in blocking solution for post *in situ* hybridization embryos. Animals were incubated with primary antibodies overnight at 4°C and then washed with PBT five times over the course of 1 h. Both larvae labeled with anti-acetylated tubulin and embryos labeled with anti-histone were then incubated for 3 h at room temperature with a 1:400 dilution of goat anti-mouse secondary antibody conjugated with the fluorescent tag Alexa Fluor 488 (Cat No. A11001, Lot No. 37977A; Molecular Probes), then washed five times over the course of 1 h with PBT. Cell membranes and larval muscles were visualized by staining with a 1:200 dilution of Alexa Fluor 488-phalloidin (Cat No. A12379, Lot No.1704534; Thermo Fisher Scientific) in block for 40 min. DNA was visualized via staining with 0.125 μg/ml Hoechst added directly to the mounting medium of 80% glycerol in 1× PBS.

### Microscopy and imaging

To optimize visualization of samples during microscopy, all specimens were equilibrated in a solution of 80% glycerol in 1× PBS. Specimens were mounted on slides and maneuvered into multiple orientations to thoroughly assess the presence of an anterior-posterior axis, dorsal-ventral axis and bilateral symmetry.

Specimens were imaged using a Zeiss LSM 710 confocal microscope (Zeiss). 3D reconstructions were generated using Fiji (Schindelin et al., 2012). All figures were constructed in Adobe Photoshop CC (version 19.0).

### Statistical analysis

Larvae were placed into one of the following treatment groups: Std-Ctrl MO, *Smad2/3* tr MO, *Smad2/3* sp MO, *Smad2/3* sp MO+*Smad2/3* mRNA, *Smad1/5/8* sp1 MO and *Smad1/5/8* sp2 MO. For statistical analyses, larvae were sorted into two categories: dorsal-ventral axis detected or no dorsal-ventral axis detected. To determine which conditions differ from each other, an omnibus Chi-square test of homogeneity, followed by post-hoc pairwise comparisons using a *z*-test of two proportions were performed. Bonferroni correction was applied to account for multiple comparisons.

## Supplementary Material

Supplementary information
