# Supplementary material for: Functional evidence that Activin/Nodal signaling is required for establishing the dorsal-ventral axis in the annelid Capitella teleta
Source: Development. 2020 Sep 23;147(18):dev189373. doi: 10.1242/dev.189373 (PMC7522025; doi:10.1242/dev.189373)
Supplement: Supplementary information [file develop-147-189373-s1.pdf]

Table S1. Detection of transcripts in individual cells of early 2d embryos

Gene detected	Total number of embryos observed	detection in cell											
		1a	1b	1c	1d	2a	2b	2c	2d	2A	2B	2C	2D
*BMP Receptor 2**	5	5	5	4	4	0	0	0	0	0	0	0	0
*SMAD1/5/8**	12	12	12	9	9	0	0	0	0	4	1	0	0
activin/inhibin/myostatin-like 5	11	11	11	10	5	0	0	0	1	3	0	0	0
*activin/inhibin/myostatin-like 4**	11	11	11	9	4	0	0	0	0	1	1	0	0
*TSG**	12	12	12	10	3	0	0	0	0	0	0	0	0
*Noggin A**	13	12	13	7	3	0	0	1	3	0	0	0	0

** denotes differentially expressed genes*

Development • Supplementary information

Table S2. TGF-beta components in *C. teleta* and corresponding protein ID numbers

TGF-beta Component*	Sequence ID
nodal	110325
BMP10	35187
BMP 5/8	172350
BMP 2/4	173895
BMP 3	184704
ADMP	184506
MAVERICK	38881
unknown bmp-like ligand	29529
MYOSTATIN	39276
activin/inhibin/myostatin-like 1 TGFβ class ligand	223591
activin/inhibin/myostatin-like 2 TGFβ class ligand	123463
activin/inhibin/myostatin-like 3 TGFβ class ligand	194641
activin/inhibin/myostatin-like 4 TGFβ class ligand	165201
activin/inhibin/myostatin-like 5 TGFβ class ligand	193459
BMP receptor 1	111904
Activin receptor 1	109715
TGFβ Receptor 1	227433
Activin Receptor 2	94926
BMP receptor 2	117843
SMAD 1/5/8	173019
SMAD 2/3	167863
SMAD 4	179368
SMAD 6/7	147083
GREMLIN	57284
Chordin Like	224618
TSG	165012
Noggin A	155479
Noggin B	207073
Follisatin	186626
Tolloid	221124
SMURF	153266
BAMBI	220461
NOMO	179513

*See Kenny et al., 2014

Development • Supplementary information
